# Reimbursement and use of oral anticoagulants during 2014–2022 - A register-based study

**DOI:** 10.1016/j.rcsop.2023.100284

**Published:** 2023-06-01

**Authors:** Emma Aarnio, Risto Huupponen, Janne Martikainen, Maarit J. Korhonen

**Affiliations:** a School of Pharmacy, University of Eastern Finland, P.O. Box 1627, 70211 Kuopio, Finland; b Institute of Biomedicine, University of Turku, FI-20014 University of Turku, Finland

**Keywords:** Oral anticoagulant, Register, Reimbursement, Treatment guideline, Atrial fibrillation

## Abstract

**Background:**

Vitamin K antagonists, warfarin in particular, have been the mainstay of anticoagulation therapy, but their use has declined in many countries since direct oral anticoagulants (DOACs) have entered the market.

**Objective:**

To examine utilization trends of oral anticoagulants (OACs) in Finland considering the reimbursement of DOACs and changes to national treatment guidelines for the treatment of atrial fibrillation (AF).

**Methods:**

Both public, aggregated data on reimbursed OAC dispensations and individual-level data on electronic dispensations during 2014–2022 were applied. Data on electronic dispensations during 2015–2016 were used to study OAC initiations. Data on entitlements to reimbursement for DOACs came from public data.

**Results:**

In 2014, there were almost 20,000 DOAC users, rising to 214,000 in 2022. The number of warfarin users declined since 2015 from over 181,000 to around 59,000 users in 2022, DOACs exceeding warfarin in the number of users in 2019. The total DOAC costs were higher than warfarin costs each year. Rivaroxaban was the most widely used DOAC during 2014–2018, and apixaban during 2019–2022. In 2015, there were more warfarin (56.7%) than DOAC (43.3%) initiators, but the result was opposite for 2016 (warfarin 39.4%, DOACs 60.6%). The number of individuals entitled to reimbursement for DOACs has increased steadily, and in 2022, there were over 196,000 individuals entitled to this reimbursement due to AF.

**Conclusions:**

The uptake of DOACs in Finland appears to have been gradual and slower than in many other countries. During the 2010s, the treatment guidelines for AF were more cautious in recommending DOACs than the European guidelines. The use of DOACs increased as their reimbursement became less restrictive.

## Highlights

• The adoption of DOACs in Finland has been slower compared to many other countries.
• The use of DOACs has increased as their reimbursement has become less restrictive.
• Treatment guidelines may also have affected prescribing of DOACs in Finland.

## 1 Introduction

Oral anticoagulants (OACs) are used in the treatment and prevention of thromboses. Common indications include atrial fibrillation (AF), pulmonary embolism (PE), deep vein thrombosis (DVT), and valvular heart diseases. Vitamin K antagonists (VKAs), warfarin in particular, have been the mainstay of anticoagulation therapy, but direct OACs (DOACs) offer an alternative in many indications.^1^ DOACs on the market include dabigatran, rivaroxaban, apixaban, and edoxaban. Another DOAC, betrixaban, is approved in the United States for the prevention of venous thromboembolism (VTE) in hospitalized patients but has been withdrawn from the market for business reasons.^1^^,^^2^ DOACs provide many advantages compared to VKAs, such as fixed dosing and no need for monitoring leading to potential savings in patients' time, travel, and monitoring costs.^3^ DOACs have been shown to be at least as effective and safer than warfarin in non-valvular AF.^4^, ^5^, ^6^, ^7^ In the European guidelines for the treatment of AF in 2012, DOACs were recommended to be considered instead of a VKA in the prevention of thromboembolism.^8^ Since the 2016 guidelines, DOACs have been preferred over VKAs.^9^^,^^10^

The adoption of DOACs has varied across and within countries. For example, in Denmark, the uptake of DOACs was rapid between 2008 and 2016.^11^ In Norway, over 50% of new OAC users started treatment with a DOAC in 2013^12^ whereas in the Valencia region in Spain, where DOACs were recommended as a second line treatment and required prior authorization, initiation with DOACs accounted only for 25% of all OAC initiations at the end of 2013.^13^ In 2015, around 16% of all OAC prescriptions in England were for DOACs with variation between regions^14^ while in Norway there were already more prevalent users of DOACs than warfarin.^12^

Treatment guidelines and reimbursement decisions affect adoption of new medicines. In Australia, the use and initiation of DOACs increased rapidly after them being listed for AF in the Pharmaceutical Benefits Scheme during 2013.^15^ Similarly, increase in DOAC prescriptions was seen in many Canadian provinces after approval for reimbursement in VTE prevention since 2009 and in AF since 2011.^16^ A Swedish study showed that the initiation of DOACs for AF was affected by regional recommendations, reimbursement decisions (2011–2013 for AF), and treatment guidelines.^17^ Furthermore, previous research in Finland on different therapeutic areas has shown that the reimbursement status of medicines influences prevalence and initiation of their use.^18^^,^^19^

Prescribing of DOACs instead of VKAs has been reported to have high budget impact in several countries following different reimbursement policies. In England, DOACs accounted for 16% of dispensed OACs and 85% of the total costs of OACs of over £190 million in 2015.^14^ In 2019, 62% of dispended OACs were DOACs that accounted for 98% of the total costs of OACs of over £537 million (6.4% of all prescription medicine costs). The annual expenditure of the Pharmaceutical Benefits Scheme on OACs in Australia increased from AUS$25.1 million in the year prior to DOACs being listed for AF to AUS$203.3 million in 2015/16.^15^ In Canada, the publicly funded expenditure on OACs increased 23–390% since approval of DOACs for reimbursement in AF in less than 4 years depending on the stringency of the providence's reimbursement mechanism.^20^ Across whole Canada, the expenditure on DOACs was CAN$180 million (accounting for 88% of OAC costs) in 2014/15.

No previous studies exist on the adoption of DOACs in Finland. In 2021, 2 DOACs were among the 10 most sold medications (according to wholesale prices) in Finland.^21^ However, there is no information on how the use of DOACs and VKAs has evolved after DOACs entering the market. Therefore, the aim was to study the trends in prevalence and incidence of OAC use in Finland considering changes in the reimbursement status of DOACs and treatment guidelines for the treatment of AF.

## 2 Methods

### 2.1 Study context

In Finland, health care is primarily publicly funded, and all Finnish residents are covered by the national health insurance.^22^ There are 3 reimbursement categories for outpatient prescription medicines: the basic rate (40% since 2016), lower special rate (65% since 2016), or higher special (100%) rate.^21^ Medicines in the special reimbursement categories are essential and meant for the treatment of severe and chronic diseases. In the 100% reimbursement category, medicines have also a corrective or substitutive effect. Pharmaceutical companies apply for reimbursability from the Pharmaceuticals Pricing Board that decides on the inclusion of medicines into the reimbursement system, reimbursable medicines' wholesale prices, and reimbursement categories.

Generally, Finnish residents receive reimbursement on all medicines belonging to the basic reimbursement category after an annual deductible of 50 euros (introduced in 2016).^21^ However, the Pharmaceuticals Pricing Board may limit the reimbursement of a new medicine to certain indications. Patients are then required to obtain an entitlement to reimbursement granted by the Social Insurance Institution (Kela) or in some cases, to have an additional note on the prescription. Special reimbursements always require an entitlement from Kela. To obtain an entitlement to reimbursement, patients need a medical certificate from their treating physician. Granted entitlements are recorded in Kela's database.

In Finland, information on prescription medicine dispensations from community pharmacies are stored in 2 different national registers. Dispensations of reimbursed prescription medicines have been recorded into the Finnish Prescription Register (FPR) since 1994.^23^ Electronic prescribing was introduced in 2010, and after a stepwise extension, became mandatory in all healthcare settings in 2017.^24^^,^^25^ Electronic prescriptions and dispensations are stored in the Prescription Centre in the Kanta database (hereafter referred to as Kanta) regardless of medicines' reimbursement status.^26^

The availability of different OACs, their reimbursement status, and the national clinical practice guidelines on AF are presented in the supplementary materials (Supplementary Material S1, Supplementary Fig. S2, Supplementary Table S3).

### 2.2 Study design and setting

National trends in OAC use were studied during 2014–2022. The study was based on all OAC purchases from Finnish community pharmacies. Prevalence of warfarin use was studied only with data from the FPR because warfarin purchases and users were better captured through reimbursed purchases than electronic dispensations during 2012–2016.^26^

According to the Finnish ethical instructions for research,^27^ this study did not require ethical approval. The National Institute of Health and Welfare granted permission to use data on electronic prescriptions and dispensations (THL/47/5.05.00/2016). Only public or de-identified data were used, and no patients were contacted.

### 2.3 Data collection

OACs were identified in registers with the Anatomical Therapeutic Chemical (ATC) codes. The ATC codes included B01AA03 (warfarin), B01AE07 (dabigatran), B01AF01 (rivaroxaban), B01AF02 (apixaban), and B01AF03 (edoxaban). Information from both public, aggregated data from the FPR (provided by Kela's statistical database, Kelasto)^28^ and individual-level data from Kanta were combined (Table 1). The aggregated data retrieved from the FPR included only reimbursed purchases (number of purchases, number of patients, total costs). From Kanta data, all electronic DOAC dispensations during 2014–2016 that were not cancelled (i.e., were valid) were determined along with identification of corrections made by Dec 31, 2016.

**Table 1:** Data applied in the study.

Study question	Study data
Prevalence of OAC use	*Warfarin:* aggregated FPR data on reimbursed purchases (2014–2022) *DOACs:* individual-level data on electronic dispensations from Kanta (2014–2016), aggregated FPR data on reimbursed purchases (2017–2022)
Incidence of OAC use	Individual-level data on electronic dispensations from Kanta (2014–2016), individual-level data on reimbursed purchases from the FPR (2014–2016)
Entitlements to restricted reimbursement for DOACs	Aggregated data from Kela (2014–2022)

DOAC: direct oral anticoagulant; FPR: the Finnish Prescription Register; OAC: oral anticoagulant.

### 2.4 Data analysis

The data was analysed with descriptive statistics using Microsoft® Excel® for Microsoft 365 MSO (Microsoft Corporation, Redmond, WA, USA). For both databases (FPR and Kanta), individuals who were dispensed an OAC at least once during a calendar year were defined as users. Annual numbers of users and total costs during 2014–2022 were calculated from aggregated FPR data or from Kanta (Table 1).

New users of OACs were determined during 2015–2016. First, the first dispensation of any OAC was determined for each individual in 2015 and 2016. Data on previous OAC purchases were retrieved both from Kanta and the FPR. Initiators were defined as individuals without any OAC purchases in the previous 365 days before the first dispensation (i.e., initiation date). If individuals were dispensed at least 2 different OACs on the initiation date, they were excluded.

For entitlements to reimbursement for DOACs, annual numbers of patients who were entitled to restricted reimbursement because of AF or DVT/PE during each year were determined.

The study is reported according to the STROBE guidelines.^29^

## 3 Results

**Fig. 1:**
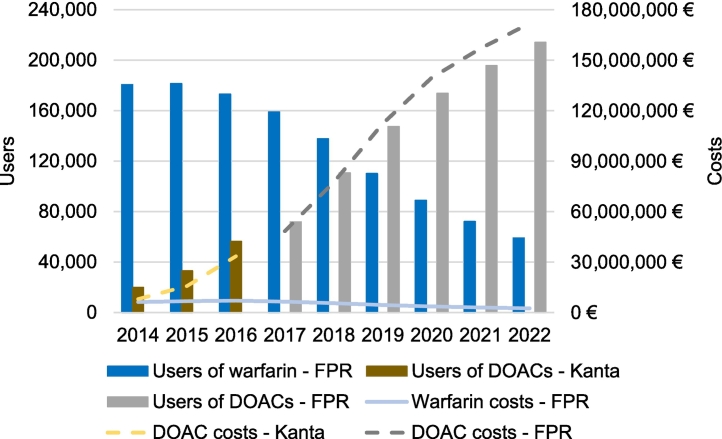
Users and total costs of oral anticoagulants in Finland. Users and costs of warfarin were identified from the Finnish Prescription Register (FPR). Users and costs of direct oral anticoagulants (DOACs) were identified from Kanta during 2014–2016 and from the FPR during 2017–2022.

The number of users of DOACs increased from almost 20,000 in 2014 to around 214,000 in 2022 (Fig. 1). Conversely, the number of warfarin users declined from over 181,000 in 2015 to around 59,000 users in 2022. Although there were less DOAC users than warfarin users until 2019, the total DOAC costs exceeded warfarin costs each year. In 2022, the total costs of DOACs were over 171 million euros while the maximum annual total costs of warfarin were 7 million euros in 2016. The total number of OAC users identified from the data increased from about 200,000 in 2014 to around 273,000 in 2022. During the same period, the total costs of OACs rose from 14 million euros to over 174 million euros.

**Fig. 2:**
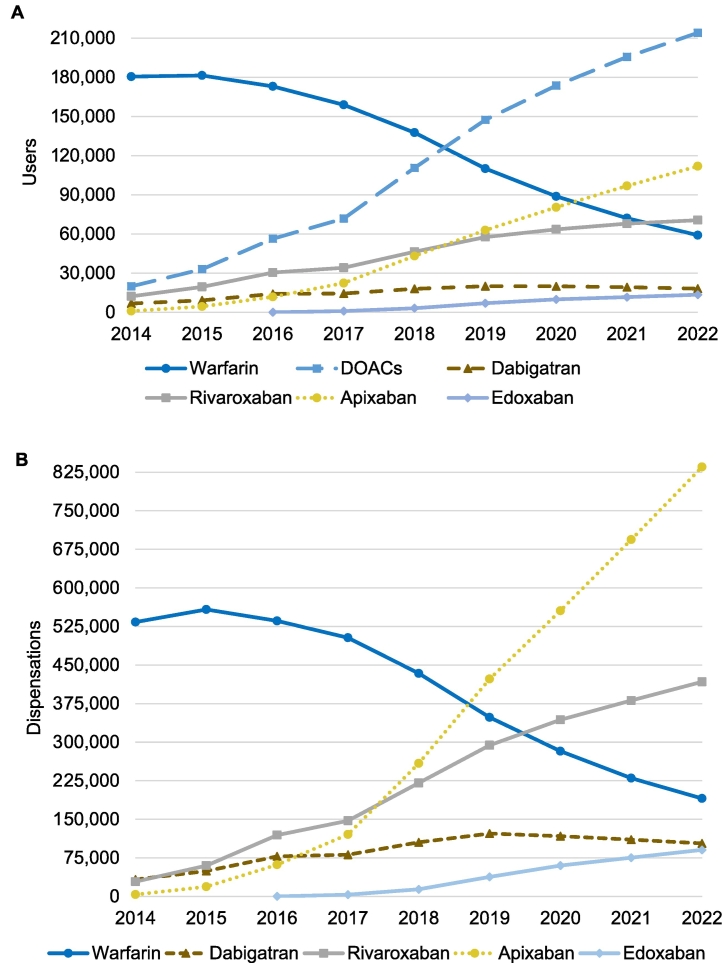
Users (A) and dispensations (B) of oral anticoagulants during 2014–2022. Data on warfarin were identified from the Finnish Prescription Register (FPR). Data on direct oral anticoagulants (DOACs) during 2014–2016 were identified from Kanta and during 2017–2022 from the FPR.

Considering each OAC separately, the number of warfarin users was observed to exceed the number of users of any specific DOAC during the study period until 2021 when apixaban was the most used OAC (Fig. 2A). Of separate DOACs, rivaroxaban had the highest number of users during 2014–2018, but during 2019–2022, apixaban was the most widely used DOAC. Apixaban was the most frequently dispensed DOAC already in 2018 (Fig. 2B). The use of dabigatran seems to have remained the same or declined slightly since 2019 while the use of all other DOACs has increased. Of DOAC users, 85.0% received lower special reimbursement due to AF in 2022. The corresponding figure for 2018 was 80.2%. During both years, users of rivaroxaban were the least likely to receive lower special reimbursement (71.4% in 2018 and 78.2% in 2022) which is in accordance with users of rivaroxaban receiving reimbursement due to DVT/PE most often (10.7% in 2022). In 2022, 57.1% of warfarin and 61.2% of DOAC users were aged 75 years or older. In this age group, 20.5% used warfarin and 44.4% apixaban, while the corresponding figures in 2019 were 44.4% and 26.5%, respectively.

**Fig. 3:**
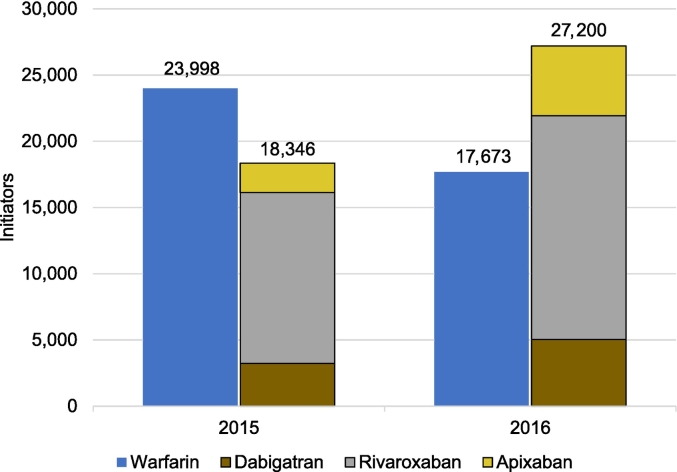
Initiators of oral anticoagulants in 2015 and 2016 based on electronic dispensations from Kanta. Edoxaban is not shown in the figure due to its low number of initiators but is calculated to the total number in 2016.

In 2015 and 2016, 42,344 and 44,873 new users of OACs were identified, respectively (Fig. 3). In addition, there were 12 new users in 2015 and 8 new users in 2016 who were dispensed 2 different OACs on the initiation date. In 2015, there were more warfarin (56.7%) than DOAC (43.3%) initiators, but the result was opposite for 2016 (39.4% vs. 60.6%, respectively). Among DOACs, users initiated most often with rivaroxaban both in 2015 (70.3%) and 2016 (62.1%). During both years, the mean age of warfarin initiators was higher than that of the patients initiating with DOACs (72.7 vs. 65.5 years in 2015, 73.2 vs. 67.3 years in 2016, respectively).

**Fig. 4:**
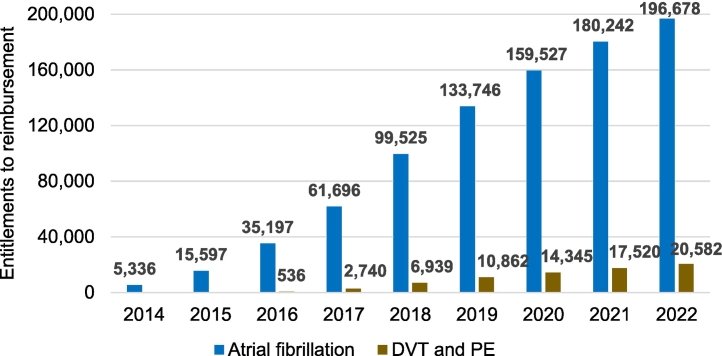
Number of people entitled to restricted reimbursement for direct oral anticoagulants based on atrial fibrillation or long-term treatment or prevention of deep vein thrombosis (DVT) and/or pulmonary embolism (PE).

The number of individuals entitled to limited reimbursement for DOACs has increased steadily (Fig. 4). Entitlements based on AF (196,000 individuals in 2022), compared to DVT/PE, are much more common. In 2022, for rivaroxaban, around 300 people were also entitled to special reimbursement due to coronary artery disease and around 550 people to basic reimbursement due to coronary artery disease or peripheral arterial disease.

## 4 Discussion

According to this register-based study in Finland, the number of warfarin users started to decline since 2015; however, there were still more warfarin than DOAC users until 2019. In the same year, apixaban became the most widely used DOAC, overtaking rivaroxaban. Initiation with warfarin was more common in 2015, but in 2016 the number of DOAC initiators exceeded the number of warfarin initiators. Despite the smaller number of users during 2014–2018, the total costs of DOACs were higher than the total costs of warfarin each year, reaching 171 million euros in 2022.

The uptake of DOACs in Finland seems slower than in other Nordic countries. In Norway, over half of new OAC users initiated with DOACs already in 2013^12^ and more Danish patients with AF initiated OAC treatment with DOACs than with warfarin in the same year.^30^ Initiating with DOACs became more likely among patients with AF in the Stockholm region, Sweden, in 2014.^17^ When looking at the prevalence of OAC use in Norway, the number of DOAC users exceeded the number of warfarin users in 2015.^12^

Also, outside the Nordic countries, DOAC use increased quickly in many countries. Patients with AF initiated OAC treatment with DOACs more often than with VKAs in France already by the end of 2012^31^ and in a large US health plan in the third quarter of 2013.^32^ Among patients initiating OAC treatment in Australia, the proportion of warfarin initiators was below 50% in September 2013.^15^ In Canada, the overall use of warfarin started to decline since February 2011 and warfarin dispensations accounted for 67% of all OAC dispensations in June 2014 with the respecting number being 99% in 2010.^16^

There are also countries where the use of DOACs can be said to have increased gradually as in Finland. In England, despite of a quick increase in initiation of DOACs, DOACs accounted for 56% of first OAC prescriptions in 2015.^33^ According to another study from England, warfarin use has declined, similarly to the now reported study, since 2015 and DOACs accounted for around 16% of all OAC prescriptions in 2015 but around 62% in 2019.^14^ Among patients with AF in the Netherlands, DOACs exceeded warfarin as the first OAC during 2016.^34^ In Czechia, warfarin accounted for around 66% of OAC utilization in 2017 with its utilization remaining relatively stable^35^ which indicates an even slower uptake than in Finland.

There are probably several reasons for the reported differences between countries' uptake of DOACs. The uptake may have been affected by local guidelines, policies, clinical practices, and reimbursement rules. The effect of reimbursement on the use of DOACs has been reported previously in many countries. In South Korea, DOACs became reimbursable in AF in January 2013 with a requirement of an opinion letter from the prescribing physician stating that warfarin is not suitable for the patient.^36^ This change affected the use of DOACs only marginally, the volume of DOAC prescriptions being around 100 prescriptions per month in the study data. However, when the requirement of the letter was removed in July 2013, the prescription volume increased immediately to 400 prescriptions per month and continued growing afterwards. After DOACs becoming reimbursable in AF, the rate of warfarin dispensations started to decrease in Australia by around 1800 dispensations per month even though the use of all OACs started to increase with a rate of around 2300 dispensations per month with DOACs explaining the difference.^15^ In Canada, the market shares of rivaroxaban and apixaban started to increase after them becoming reimbursable.^16^ In addition, the use of DOACs has varied in Canada with the use increasing more in provinces with less stringent reimbursement mechanism.^20^ Previous studies from the US and Denmark have also reported that patients' lower socioeconomic status is associated with lower probability of receiving a DOAC.^32^^,^^37^, ^38^, ^39^ All these previous studies show that cost is an important factor in access to DOACs.

Other factors have also been reported to affect the use of DOACs. Publication of the European guidelines for the treatment of AF in 2012 was associated with an increase in the initiation of DOACs in the Stockholm region.^17^ In addition, changes in the regional recommendations were associated with the choice between different DOACs in AF. In Canada, rivaroxaban was the first DOAC to receive authorities' recommendation for VTE prevention after major orthopaedic surgery, leading to it gaining a substantial portion of the market share in this indication.^16^ In addition to changes in the use of DOACs around times of changes in their reimbursement, the use of DOACs temporarily decreased after security warnings issued by health authorities in France.^31^ However, even though French health authorities did not recommend DOACs as initial OAC treatments in AF, the uptake of DOACs was quick.

In Finland, the quality of warfarin treatment, measured as time in therapeutic range, has been considered good compared to many non-Nordic countries^40^, ^41^, ^42^ and this may have attenuated clinicians' drive to change ongoing warfarin treatment or start new patients with DOACs. Unlike warfarin, DOACs had no specific reversal agent at the time of their launch; reversal agents for DOACs (idarucizumab and andexanet alfa) became available during the latter half of 2010s^43^ which may have also hindered the uptake of DOACs. The reimbursement status and criteria for DOACs and the Current Care Guidelines for AF changed many times in Finland during the study period. According to the data, the largest increase in the number of DOAC users (around +250%) occurred in 2018 when there was no update in the Current Care Guidelines, but DOACs became reimbursable at the lower special rate. Unfortunately, no definite conclusions can be drawn based on this notion as the data included only reimbursed purchases in 2017 and 2018. The real increase in the number of users may not have been so drastic due to the possibility that patients who purchased DOACs without reimbursement in 2017 received entitlement to reimbursement in 2018. However, a previous study where physicians reported on how patients' views on medicine co-payment influence the choice of an OAC^44^ supports the notion of a larger role of reimbursement than of treatment guidelines in the use of DOACs in Finland. In addition, the use of DOACs exceeded the use of warfarin in 2019 although DOACs were preferred over warfarin for new users (i.e., not only for short-term treatment) not until the latest update of the treatment guidelines for AF in 2021.

Overall, the Finnish approach to DOACs seems more cautious than in many other countries. The Current Care Guideline for AF has recommended a patient-specific choice between warfarin and DOACs since the introduction of DOACs in the guidelines in 2012. In 2015, the guidelines recommended DOACs as the primary choice for short-term treatment. As the primary choice for new OAC treatment, DOACs were recommended in the 2021 update. This is in contrast to the European guidelines that recommended DOACs to be considered for most patients with AF in 2012^8^ and have preferred DOACs over VKAs already since 2016.^9^^,^^10^

This study is not without limits. Firstly, the data covered only reimbursed DOAC purchases since 2017 and, therefore, the real use of DOACs may have been higher than shown in the study. However, the reimbursement of DOACs became less restrictive in 2017, leading the FPR likely to cover DOAC purchases better than in the previous years. Secondly, patients receiving only paper prescriptions for OACs are missing from the applied data on DOACs during 2014–2016 and on OAC initiation during 2015–2016. Therefore, it is possible that 2016 is not the year DOACs overtook warfarin in OAC initiations. In 2015, however, over 90% of prescriptions dispensed in Finnish pharmacies were electronic^45^ suggesting that most of OAC dispensations were covered by the data. Thirdly, no statistical analyses were conducted, such as interrupted time series analysis, to study the effect of changes in the reimbursement of DOACs and in the guidelines for the treatment of AF on the use of OACs. The changes were so frequent that the effect of a single change would have been difficult to discern. Fourthly, the data did not include information on the indications of OAC treatment which prevented studying the possible variations in the use of OACs by indication. Indications recorded in the electronic OAC prescriptions written in 2016 have been previously examined and information on indication was omitted from every third DOAC prescription and from 2/3 of warfarin prescriptions.^26^ Based on entitlements to reimbursement and the reimbursed purchases from 2019 to 2022, the most common indication for DOACs was AF. Because no entitlement was required for the reimbursement of warfarin, comparison between warfarin and DOACs by indications, unfortunately, was not possible.

## 5 Conclusion

Adoption of new pharmacotherapies for common diseases is a challenge for the medical community and the reimbursement system. The Finnish experience from DOACs show that their use seems to have increased as their reimbursement has become less restrictive, indicating the importance of co-payment in their uptake. Despite the several updates of the Finnish treatment guidelines, the reimbursement status and criteria of DOACs seem to have had a greater effect on their use than treatment guidelines and, therefore, have steered the use of DOACs. Overall, the uptake of DOACs appears to have been gradual and slower in Finland than in many other countries.

## Funding statement

This work was supported by the Social Insurance Institution of Finland (24/26/2015). The funder had no role in the design and conduct of the study, analysis and interpretation of the data nor in the decision to submit the manuscript for publication. EA received funding also from the 10.13039/501100003125Finnish Cultural Foundation and MJK from the 10.13039/501100009420Hospital District of Southwest Finland.

## CRediT authorship contribution statement

**Emma Aarnio:** Conceptualization, Formal analysis, Investigation, Writing – original draft, Visualization, Funding acquisition. **Risto Huupponen:** Writing – review & editing, Funding acquisition. **Janne Martikainen:** Resources, Writing – review & editing. **Maarit J. Korhonen:** Conceptualization, Writing – review & editing, Supervision, Funding acquisition.

## Declaration of Competing Interest

The authors declare the following financial interests/personal relationships which may be considered as potential competing interests:

Emma Aarnio reports a relationship with Merck & Co that includes: consulting or advisory and speaking and lecture fees; a relationship with UCB Pharma that includes: speaking and lecture fees; a relationship with Alk-Abello Nordic that includes: speaking and lecture fees. Janne Martikainen reports a relationship with ESiOR Oy that includes: equity or stocks. Maarit J Korhonen reports a relationship with Oriola Finland Oy that includes: consulting or advisory. Risto Huupponen is the past chairman of the Expert Panel of Meds 75+-database (drug treatment in older subjects) and a member of the Expert Panel for classification of high-risk medicines, both at the Finnish Medicines Agency.

## Appendix A Supplementary data

Supplementary material 1 Description of oral anticoagulants and the clinical practice guidelines on atrial fibrillation in Finland. Supplementary material 1

Supplementary material 2 Changes in the reimbursement status of direct oral anticoagulants in Finland. Supplementary material 2

Supplementary material 3 Choice of an oral anticoagulant in the Current Care Guidelines for atrial fibrillation with some important points and changes highlighted. Supplementary material 3
