# Supplementary material for: Reimbursement and use of oral anticoagulants during 2014–2022 - A register-based study
Source: Explor Res Clin Soc Pharm. 2023 Jun 1;11:100284. doi: 10.1016/j.rcsop.2023.100284 (PMC10393798; doi:10.1016/j.rcsop.2023.100284)
Supplement: Supplementary material 1 — Description of oral anticoagulants and the clinical practice guidelines on atrial fibrillation in Finland. [file mmc1.pdf]

Supplementary Material S1. Description of oral anticoagulants and the clinical practice guidelines on atrial fibrillation in Finland.

OACs in Finland

Warfarin, dabigatran, rivaroxaban, apixaban, and edoxaban are the currently available OACs in Finland. Dabigatran and rivaroxaban were the first DOACs to enter the market in the European Union (EU), including Finland, in 2008 to be used in the prevention of VTE after elective hip or knee replacement surgery.[1,2] In the EU, extensions of marketing approval to include non-valvular AF was first granted to dabigatran (August 2011),[3] followed by rivaroxaban[4] and apixaban.[5] Edoxaban entered the market in 2015 and was directly approved for the treatment of non-valvular AF.[6]

All warfarin products are reimbursable at the basic rate without any restrictions.[7] The reimbursement of DOACs is, however, multifaceted. There were several changes in the reimbursement status of DOACs during the 2010s depending on the DOAC, indication and severity of disease (Supplementary Figure S2). To receive reimbursement for DOACs in short-term treatment, an additional note on the prescription is required. In long-term treatment, the patient needs an entitlement from Kela. The criteria for this entitlement changed several times during the 2010s.

DOACs were first reimbursable at the basic rate for prevention of VTE after elective hip or knee replacement surgery starting 2009 (Supplementary Figure S2). In 2012, dabigatran and rivaroxaban, followed by apixaban in 2013, became restrictedly reimbursable at the basic rate in the treatment of non-valvular AF. Criteria for this entitlement were at least medium stroke risk ($CHA_2DS_2$-VASc score ≥1) and failed or contraindicated warfarin treatment. After changes in these criteria, becoming restrictedly reimbursable at the basic rate in the short-term treatment of patients undergoing cardioversion, and edoxaban becoming reimbursable in the treatment of AF in 2016, all DOACs became restrictedly reimbursable at the lower special rate in the long-term treatment of AF in 2018.

Rivaroxaban, in 2012, was the first DOAC to be restrictedly reimbursable at the basic rate for the short-term treatment (<6 months) of DVT and prevention of DVT or PE (Supplementary Figure S2). In 2013, it was also reimbursable in the short-term treatment of PE. In 2015, the reimbursement rules for dabigatran and apixaban became similar as for rivaroxaban. All four DOACs became restrictedly reimbursable at the basic rate in the long-term prevention of DVT and PE in 2016. The criteria for the entitlement to this reimbursement chanced several times during 2017–2018.

During the 2010s, the price of warfarin was around 8–11 euros per 100 tablets (without reimbursement).[8] When becoming reimbursable in the treatment of AF in 2012, the price of 1 month's dose of dabigatran and rivaroxaban was around 100 euros. In February 2023, the price of 1 month's dose of DOACs was around 80 euros. For patients entitled to reimbursement at the lower special rate, co-payment for 1 month's dose of DOACs would have been little below 30 euros. The price of warfarin has remained the same, the co-payment for 100 tablets being around 6–8 euros.

Current Care Guidelines on atrial fibrillation

In Finland, the Finnish Medical Society Duodecim in association with various medical specialist associations develops national clinical practice guidelines called the Current Care Guidelines.[9] The Current Care Guideline for AF was introduced in 2005 and has been updated 6 times (Supplementary Table S3).[10] Since 2012, the guideline has recommended patient-specific selection between warfarin and DOACs based on the evaluation of medicines' properties and the patient's opinion.[11] In 2012, it was stated that there is not enough evidence to justify a wide transition towards the use of DOACs but that they are a good alternative. In 2014, DOACs were stated to be a good choice, for example, in short-term treatment and for many new patients.[12] Since 2015, DOACs have been the primary choice in short-term treatment.[13] In the update in 2017, DOACs were stated to be a good choice for new patients.[14] The most recent update for the guidelines was published in 2021, and DOACs are now preferred for most new patients.[15] In all updates, the regular contacts to health care related to the monitoring of warfarin treatment have been seen potentially useful for patients with multimorbidity and older patients.

REFERENCES

1. Lippi G, Mattiuzzi C, Cervellin G, Favaloro EJ. Direct oral anticoagulants: analysis of worldwide use and popularity using Google Trends. Ann Transl Med 2017;5:322. https://doi.org/10.21037/ATM.2017.06.65

2. Haastrup SB, Hellfritzsch M, Rasmussen L, Pottegård A, Grove EL. Use of non-vitamin K antagonist oral anticoagulants 2008–2016: A Danish nationwide cohort study. Basic Clin Pharmacol Toxicol 2018;123:452–463. https://doi.org/10.1111/bcpt.13024

3. European Medicines Agency. Committee for Medicinal Products for Human Use. CHMP Assessment Report. EMA/CHMP/203468/2011. www.ema.europa.eu Accessed February 21, 2023.

4. European Medicines Agency. Committee for Medicinal Products for Human Use. Summary of Opinion for Xarelto. EMA/CHMP/753436/2011. www.ema.europa.eu Accessed February 21, 2023.

5. European Medicines Agency. Committee for Medicinal Products for Human Use. Assessment Report of Eliquis. EMA/641505/2012. www.ema.europa.eu Accessed February 21, 2023.

6. European Medicines Agency. Committee for Medicinal Products for Human Use. Summary of Opinion for Lixiana. EMA/CHMP/239353/2015. www.ema.europa.eu Accessed February 21, 2023.

7. The Social Insurance Institution of Finland. Medicinal Products Database. https://asiointi.kela.fi/laakekys_app/LaakekysApplication?kieli=en Accessed February 21, 2023.
8. Pharmaceuticals Pricing Board. Reimbursable authorized medicinal products and their prices. https://www.hila.fi/en/notices/reimbursable-authorized-medicinal-products-and-their-prices/ Accessed February 21, 2023.
9. The Finnish Medical Society Duodecim. About Current Care Guidelines. https://www.kaypahoito.fi/en/about-current-care-guidelines; 2022. Accessed February 21, 2023.
10. Atrial fibrillation. Current Care Guidelines. Working group set up by the Finnish Cardiac Society. Duodecim 2005;121:2470–2494.
11. Atrial fibrillation. Current Care Guidelines. Working group set up by the Finnish Medical Society Duodecim and the Finnish Cardiac Society. Helsinki: The Finnish Medical Society Duodecim; 2012.
12. Atrial fibrillation. Current Care Guidelines. Working group set up by the Finnish Medical Society Duodecim and the Finnish Cardiac Society. Helsinki: The Finnish Medical Society Duodecim; 2014.
13. Atrial fibrillation. Current Care Guidelines. Working group set up by the Finnish Medical Society Duodecim and the Finnish Cardiac Society. Helsinki: The Finnish Medical Society Duodecim; 2015.
14. Atrial fibrillation. Current Care Guidelines. Working group set up by the Finnish Medical Society Duodecim and the Finnish Cardiac Society. Helsinki: The Finnish Medical Society Duodecim; 2017.
15. Atrial fibrillation. Current Care Guidelines. Working group set up by the Finnish Medical Society Duodecim and the Finnish Cardiac Society. Helsinki: The Finnish Medical Society Duodecim; 2021. Available online at: www.kaypahoito.fi.
