# Supplementary material for: Reimbursement and use of oral anticoagulants during 2014–2022 - A register-based study
Source: Explor Res Clin Soc Pharm. 2023 Jun 1;11:100284. doi: 10.1016/j.rcsop.2023.100284 (PMC10393798; doi:10.1016/j.rcsop.2023.100284)
Supplement: Supplementary material 2 — Changes in the reimbursement status of direct oral anticoagulants in Finland. [file mmc2.pdf]

Supplementary Figure 2. Changes in the reimbursement status of direct oral anticoagulants in Finland

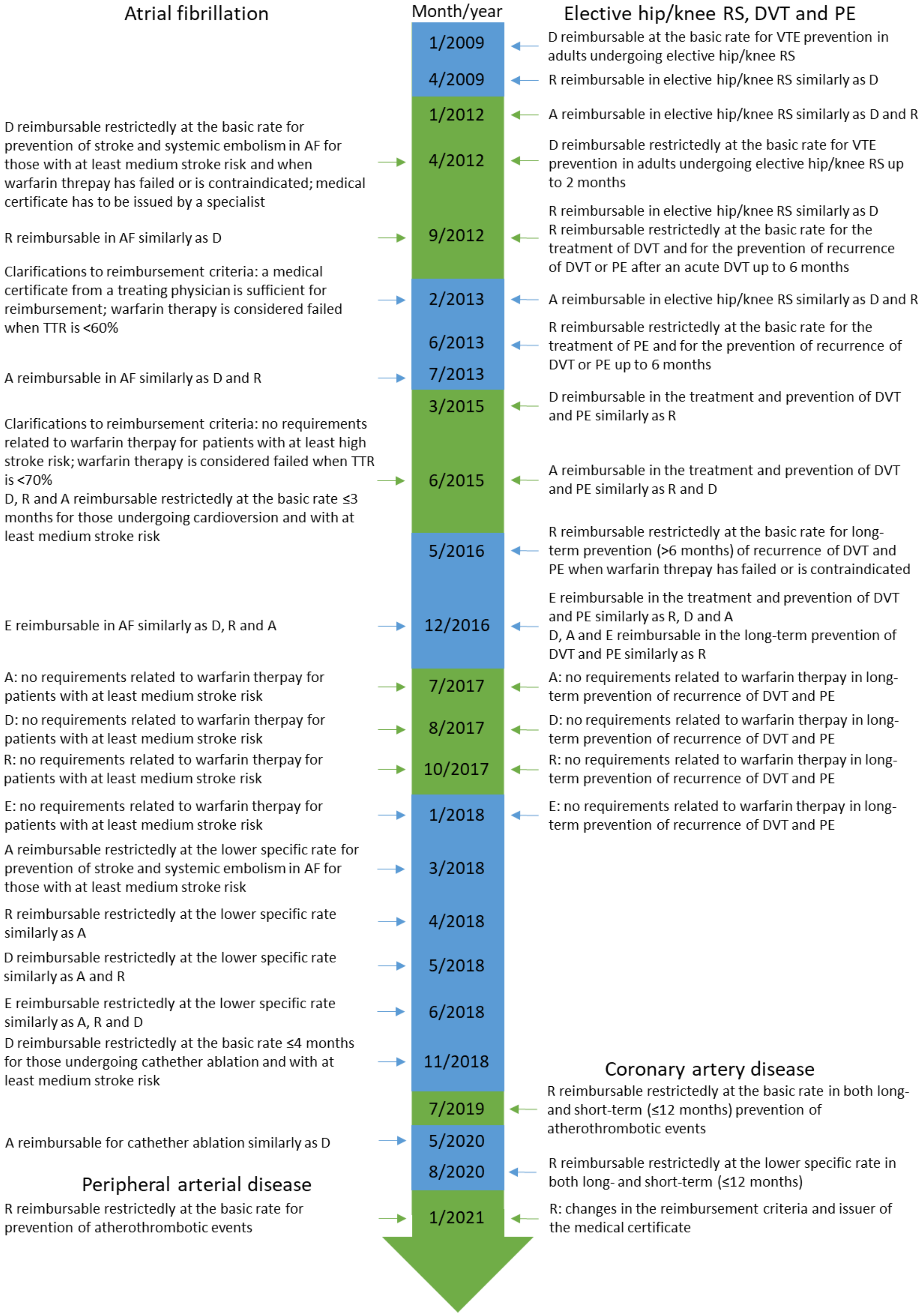

A: apixaban, AF: atrial fibrillation, D: dabigatran, DVT: deep vein thrombosis, E: edoxaban, PE: pulmonary embolism, R: rivaroxaban, RS: replacement surgery, TTR: time in therapeutic range, VTE: venous thromboembolism
