# Supplementary material for: Reimbursement and use of oral anticoagulants during 2014–2022 - A register-based study
Source: Explor Res Clin Soc Pharm. 2023 Jun 1;11:100284. doi: 10.1016/j.rcsop.2023.100284 (PMC10393798; doi:10.1016/j.rcsop.2023.100284)
Supplement: Supplementary material 3 — Choice of an oral anticoagulant in the Current Care Guidelines for atrial fibrillation with some important points and changes highlighted. [file mmc3.pdf]

Supplementary Table 3. Choice of an oral anticoagulant in the Current Care Guidelines for atrial fibrillation with some important points and changes highlighted

| | |
|---|---|
| 2005 | - **Warfarin is recommended for stroke prevention** for all patients with AF except for under 60-year-olds with a small risk (<2%/year)<br>- ASA suitable for stroke prevention for patients with a small risk and it can be an alternative for warfarin for patients with a medium risk (2–4%/year) |
| 2011 | - Warfarin is recommended for stroke prevention for all patients with AF except for those with a small risk (<1%/year)<br>- The benefits of ASA for patients with a small risk are minor and no antithrombotic treatment is usually needed, but in some special cases it can be an alternative for warfarin for patients with a medium risk (1–2%/year) |
| 2012 | - **The quality of warfarin treatment in Finland is seen on average to be good and there is said to be not enough evidence to justify a wide transition towards the use of DOACs**<br>- The **choice between warfarin and DOACs is recommended to be patient-specific** and to be based on the advantages (e.g., more convenient, less intracranial haemorrhages) and disadvantages (e.g., lack of antidote, price) of DOACs as well as on patient’s wishes<br>- No need to switch warfarin treatment with good quality<br>- DOACs are a good alternative when warfarin is not suitable or when there are problems with monitoring of warfarin treatment<br>- DOACs are more convenient for patients of working-age<br>- **Older patients and patients with multiple morbidities possibly benefit from regular contacts to healthcare related to monitoring of warfarin treatment**<br>- DOACs cause less intracranial haemorrhages than warfarin, but should not be used for patients that have a higher bleeding than stroke risk<br>- Clinical experience with rivaroxaban is minor<br>- DOACs have not been compared and no comment can be given for the choice between them |
| 2014 | - **Patient-specific choice between warfarin and DOACs**<br>- **DOACs** (dabigatran, rivaroxaban, apixaban) **are at least as efficient and safer than warfarin in stroke prevention**<br>- Warfarin treatment is efficient and safe when TTR is ≥70%<br>- Older and multimorbid patients may benefit from monitoring of warfarin treatment, DOACs more convenient for patients of working-age<br>- **DOACs are a good choice**<br>○ **for short-term treatment**<br>○ if warfarin treatment is not suitable<br>○ when there are problems with monitoring of warfarin<br>○ if TTR is <70% despite of good adherence<br>○ **for many new patients**<br>- No need to switch warfarin treatment with good quality<br>- No comment can be given for the choice between DOACs<br>- ASA is not recommended for stroke prevention |
| 2015 | - Patient-specific choice between warfarin and DOACs<br>- DOACs (dabigatran, rivaroxaban, apixaban) are at least as efficient and safer than warfarin in stroke prevention<br>- DOACs are a good choice for many new patients because of their convenience and better adherence to them<br>- Older and multimorbid patients may benefit from monitoring of warfarin treatment |

*(continued)*

| | |
|---|---|
| 2015 | - **In short-term treatment, DOACs are the primary choice**<br>- In long-term treatment, good quality warfarin treatment can be continued but a prompt switch to a DOAC is necessary if<br>○ warfarin treatment cannot be continued (e.g., adverse effects)<br>○ there are problems with monitoring of warfarin treatment<br>○ TTR is <70% despite of good adherence<br>- No comment can be given for the choice between DOACs |
| 2017 | - Patient-specific choice between DOACs and warfarin<br>- DOACs (dabigatran, rivaroxaban, apixaban, edoxaban) are at least as efficient and safer than warfarin in stroke prevention<br>- Based on new research, **the efficacy and safety of warfarin treatment is better when TTR is ≥80%** (compared to the previous limit of 70%)<br>- DOACs are a good choice for new patients because of their convenience, safety, and better adherence to them<br>- **DOACs** cause less intracranial haemorrhages and are, therefore, a **safer choice for patients who are at a greater risk of intracranial haemorrhages**<br>- Older and multimorbid patients may benefit from monitoring of warfarin treatment<br>- In long-term treatment, good quality warfarin treatment can be continued but a prompt switch to a DOAC is necessary if<br>○ warfarin treatment cannot be continued (e.g., adverse effects)<br>○ there are problems with monitoring of warfarin<br>- If TTR <80%, an assessment is needed whether warfarin treatment needs optimizing or a switch to a DOAC is required<br>- In short-term treatment, DOACs are the primary choice<br>- If a patient with a bioprosthetic valve does not require warfarin treatment, a DOAC can be used<br>- No comment can be given for the choice between DOACs |
| 2021 | - Patient-specific choice between DOACs and warfarin<br>- DOACs (dabigatran, rivaroxaban, apixaban, edoxaban) are at least as efficient and safer than warfarin in stroke prevention<br>- **DOACs are the primary choice for most new patients** because of their safety and better adherence to them<br>- DOACs cause less intracranial haemorrhages and are, therefore, a safer choice for patients who are at a greater risk of intracranial haemorrhages<br>- Older and multimorbid patients may benefit from monitoring of warfarin treatment<br>- In long-term treatment, good quality warfarin treatment can be continued but a prompt switch to a DOAC is necessary if<br>○ warfarin treatment cannot be continued (e.g., adverse effects)<br>○ there are problems with monitoring of warfarin<br>- If TTR <80%, an assessment is needed whether warfarin treatment needs optimizing or a switch to a DOAC is required<br>- In short-term treatment, DOACs are the primary choice<br>- If a patient with a bioprosthetic valve does not require warfarin treatment, a DOAC can be used<br>- Specific antidote available for all DOACs<br>- DOACs have not been compared and no comment can be given for the choice between them |

AF: atrial fibrillation, ASA: acetylsalicylic acid, DOAC: direct oral anticoagulant, TTR: time in therapeutic range
